# β-Lactamase Characterization of Gram-Negative Pathogens Recovered from Patients Enrolled in the Phase 2 Trials for Ceftazidime-Avibactam: Clinical Efficacies Analyzed against Subsets of Molecularly Characterized Isolates

**DOI:** 10.1128/AAC.01173-15

**Published:** 2016-02-26

**Authors:** Rodrigo E. Mendes, Mariana Castanheira, Leanne Gasink, Gregory G. Stone, Wright W. Nichols, Robert K. Flamm, Ronald N. Jones

**Affiliations:** a JMI Laboratories, North Liberty, Iowa, USA; b AstraZeneca Pharmaceuticals LP, Waltham, Massachusetts, USA

## Abstract

The correlation of the clinical efficacies of ceftazidime-avibactam and comparators (carbapenems) was evaluated against baseline Gram-negative isolates having characterized β-lactam resistance mechanisms from complicated urinary tract infection (cUTI) and complicated intra-abdominal infection (cIAI) phase 2 trials. Enterobacteriaceae displaying ceftriaxone and/or ceftazidime MICs of ≥2 μg/ml (69 isolates) and nonfermentative Gram-negative bacilli (NF-GNB [three isolates]) with ceftazidime MICs of ≥16 μg/ml were characterized for their narrow- and extended-spectrum β-lactamase (ESBL) content. Enterobacteriaceae (one isolate) and NF-GNB (three isolates) with imipenem/meropenem MICs of ≥2 and ≥16 μg/ml, respectively, were tested for carbapenemases. All cUTI E. coli had the lineage background investigated (ST131-like versus non-ST131-like). The primary efficacy endpoint was microbiological response (eradication) at test of cure (TOC) for cUTI and clinical response (inferred microbiological eradication) at TOC for cIAI. A total of 34.1% of baseline cUTI (36.4%) and cIAI (33.1%) pathogens met the MIC-based screening criteria (screen positive). All screen-positive cUTI pathogens were CTX-M-producing E. coli, except for one E. cloacae isolate with AmpC overexpression. The majority (66.7%) of screen-positive cIAI isolates produced CTX-M-type coupled with a diverse array of other β-lactamases. Similar favorable responses were observed with ceftazidime-avibactam (93.3%) and carbapenems (90.9%), when a non-ESBL Enterobacteriaceae isolate was recovered at the baseline visit. When an ESBL Enterobacteriaceae isolate was present, the favorable responses were 85.7% and 80.0% with ceftazidime-avibactam and carbapenems, respectively. Higher favorable responses were observed with ceftazidime-avibactam (75.0%) than with carbapenems (66.7%) when an ST131-like E. coli isolate was recovered at baseline, as when a non-ST131-like isolate was present (93.8% versus 86.7%, respectively). The efficacy of ceftazidime-avibactam was similar to that of carbapenems for treatment of cUTI and cIAI caused by ESBL organisms.

## INTRODUCTION

*Enterobacteriaceae* are a common cause of community-acquired and health care-acquired infections, with Escherichia coli, Klebsiella spp. and Enterobacter spp. among the most common organisms (1). Antimicrobial resistance among Enterobacteriaceae mostly reflects the worldwide emergence and dissemination in the late 1980s of extended-spectrum β-lactamases (ESBLs), such as *bla*_TEM_ and *bla*_SHV_ allelic variants (1). However, the epidemiology of these isolates changed dramatically during early years of 2000s, and TEM and SHV ESBL-encoding genes have slowly been replaced by *bla*_CTX-M_ genes, which have been detected in community- and hospital-acquired Enterobacteriaceae (2–4). In 2012, 10.1% (581/5,739) of E. coli, Klebsiella, and Proteus mirabilis isolates from U.S. hospitals were found to carry ESBL genes, and the majority (61.6%) of those were *bla*_CTX-M_ (5).

During the late 1990s, carbapenem-resistant Enterobacteriaceae (CRE) began to emerge (6). In 2012, 4.6% of acute care hospitals reported at least one CRE isolate, and the proportion of Enterobacteriaceae that were CRE increased from 1.2% in 2001 to 4.2% in 2011 in the National Nosocomial Infection Surveillance system (NNIS) and the National Healthcare Safety Network (NHSN) and from 0% in 2001 to 1.4% in 2010 in the Surveillance Network–USA (TSN) (1). Overall, the majority of CRE isolates in the United States harbor a Klebsiella pneumoniae carbapenemase (KPC) serine carbapenemase-encoding gene (6). *bla*_KPC_ genes are mostly detected in Klebsiella pneumoniae, but they have been observed in numerous Enterobacteriaceae species and have become endemic in several hospitals worldwide (7).

CRE isolates often demonstrate a susceptible phenotype to polymyxin B compounds and tigecycline only (8) and correlate significantly to the patients' degree of morbidity (9). Thus, the use of broad-spectrum β-lactamase inhibitor compounds in combination with β-lactam agents is a promising option in development for treatment of infections caused by ESBL and carbapenemase producers (10). Avibactam is a novel non-β-lactam β-lactamase inhibitor of β-lactam-hydrolyzing enzymes belonging to Ambler structural classes A and C, as well as some class D enzymes (10). Previous phase 2 clinical trials demonstrated the efficacy, safety, and tolerability of ceftazidime-avibactam versus comparator agents for treatment of complicated urinary tract infections (cUTI) and complicated intra-abdominal infections (cIAI) (11, 12). The present study characterized the β-lactamase genes in baseline pathogens recovered during those phase 2 trials. In addition, this study correlates the efficacy of ceftazidime-avibactam and comparators against subsets of isolates harboring β-lactam resistance mechanisms.

## MATERIALS AND METHODS

### Patients, clinical isolates, study treatment, and endpoints.

Male and female patients between the ages of 18 and 90 years were enrolled in the phase 2 clinical trials for ceftazidime-avibactam (clinicaltrials.gov identifiers NCT00752219 and NCT00690378) (11, 12). Hospitalized patients were enrolled from medical centers located in Guatemala, India, Jordan, Lebanon, and the United States for the cUTI trial and Bulgaria, France, India, Lebanon, Poland, Romania, Russia, and the United States for the cIAI trial.

Patients eligible for the cUTI trial were stratified by diagnosis (acute pyelonephritis or other cUTI) and randomized 1:1 to 0.5 g ceftazidime–0.125 g avibactam (here, “0.5/0.125 g”) (intravenous [i.v.] every 8 h) or imipenem-cilastatin (0.5 g i.v. every 6 h). Oral ciprofloxacin (0.5 g twice daily) or alternative oral therapy was permitted after at least 4 days of initial i.v. therapy. Pathogens were recovered via urine culture at the baseline (the first one being termed “baseline”) and follow-up visits. Blood cultures were performed when clinically indicated or in patients with indwelling catheters and stents. The primary efficacy endpoint was microbiological response at test of cure (TOC), 5 to 9 days after end of treatment in the microbiologically evaluable (ME) population. Clinical response was a secondary endpoint.

For cIAI, eligible patients with a presumed (preoperative) or definitive (intraoperative or postoperative) diagnosis of cIAI were randomized 1:1 to ceftazidime-avibactam (2/0.5 g i.v. every 8 h) and metronidazole (0.5 g i.v. every 8 h) or meropenem (1 g i.v. every 8 h) plus placebo. Isolates were obtained during the study qualifying operative procedure (i.e., laparotomy, laparoscopy, or percutaneous drainage) and during any subsequent operative procedures. Blood cultures were also collected from all patients at the baseline visit. The primary efficacy endpoint was clinical response at TOC, 2 weeks after the end of treatment in the ME population. The ME population was defined as clinically evaluable patients with a confirmed cUTI or cIAI, at least one baseline pathogen susceptible to the study therapy agents, who received treatment, and who had a clinical assessment at the TOC visit (see references 11 and 12 for additional information).

For the cUTI trial, a favorable response was defined as eradication of all pathogens from urine (<10^4^ CFU/ml) and blood, while in the cIAI trial, a favorable response was defined as complete resolution or significant improvement of signs/symptoms of infection with no requirements for additional antimicrobial therapy (11, 12).

### Selection criteria for β-lactamase screen and analysis.

All baseline isolates were centrally tested for susceptibility by broth microdilution (Clinical and Laboratory Standards Institute [CLSI] standard M07-A9, 2012) (13). Enterobacteriaceae displaying ceftriaxone and/or ceftazidime MIC results of ≥2 μg/ml and nonfermentative Gram-negative bacilli (NF-GNB) with ceftazidime MIC results of ≥16 μg/ml were selected for further characterization of narrow- and extended-spectrum β-lactamase (ESBL) genes. Enterobacteriaceae and NF-GNB isolates exhibiting imipenem/meropenem MIC results of ≥2 and ≥16 μg/ml, respectively, were tested for the presence of carbapenemase-encoding genes, as previously described (14–17).

Isolates that met the MIC screening criteria described above were subjected to a microarray-based assay, the Check-MDR CT101 kit, according to the manufacturer's instructions (Check-Points, Wageningen, Netherlands). This kit has the capabilities to detect CTX-M groups 1, 2, 8 + 25, and 9, non-ESBL and ESBL variants of TEM and SHV, plasmid AmpC (ACC, ACT/MIR, CMY, DHA, and FOX), and KPC- and NDM-encoding genes (14). Supplemental multiplex PCR assays were utilized to detect additional ESBL-encoding genes (*bla*_GES_, *bla*_VEB_, *bla*_PER_, and oxacillinase enzyme genes [*bla*_OXA-2_, *bla*_OXA-10_, and *bla*_OXA-13_ groups, *bla*_OXA-18_, and *bla*_OXA-45_]) (14) and carbapenemase-encoding genes (*bla*_IMP_, *bla*_VIM_, *bla*_NDM-1_, *bla*_OXA-48_, *bla*_GES_, *bla*_NMC-A_, *bla*_SME_, and *bla*_IMI_) (15). Acinetobacter species isolates were also screened for the *bla*_OXA-23_, *bla*_OXA-24_, and *bla*_OXA-58_ groups (16). All results obtained by the Check-Points and supplemental PCR assays were confirmed by single PCRs and subsequent determination of the gene allele by sequencing analysis of both strands (Sanger method); nucleotide and amino acid sequences were analyzed using the Lasergene software package (DNASTAR, Madison, WI). Amino acid sequences were compared with those available through the internet using BLAST (http://www.ncbi.nlm.nih.gov/blast/).

The transcription levels of the chromosomally encoded AmpC were determined in Pseudomonas aeruginosa, E. coli, and Enterobacter cloacae. The transcription levels of the chromosomal *ampC* gene were determined by the quantification of the target gene mRNA using a normalized expression analysis method and relative comparison to susceptible control strains (18, 19). A given strain was considered to overexpress the *ampC* gene when at least a 10-fold greater difference of *ampC* transcripts was detected compared with a species-specific wild-type reference control strain.

### Determination of E. coli strains associated with ST131 and PFGE.

All E. coli strains recovered during the cUTI trial were investigated by pulsed-field gel electrophoresis (PFGE) for the presence of two single nucleotide polymorphisms (SNPs), namely, thymine 144 and adenine 450 in the *pabB* gene, as previously described by Dhanji et al. (20). The presence of these two SNPs is known to be unique to sequence type 131 (ST131) strains, and they were described here as “ST131-like.”

## RESULTS

### Study population.

A total of 192 ME patients were included in the study, with 26 and 20 ME patients included in the ceftazidime-avibactam and carbapenem arms, respectively, for the cUTI trial, while 60 and 70 patients, respectively, were included in the arms for the cIAI trial (11, 12). Most ME patients had a single baseline aerobic Gram-negative pathogen recovered during the screening visit, except for four (4/62 [6.5%]) patients in the cUTI trial and 14 (14/130 [10.8%]) patients in the cIAI trial who had multiple aerobic Gram-negative organisms recovered at the baseline visit (see Tables 2 and 3).

### Antimicrobial susceptibility profiles.

Table 1 lists the MIC values obtained for baseline isolates recovered from ME patients enrolled in both the cIAI and cUTI trials. Baseline Enterobacteriaceae isolates recovered from cIAI demonstrated a bimodal MIC distribution when tested against ceftazidime, with one modal MIC of 0.12 μg/ml and a second mode at >32 μg/ml. Avibactam restored the ceftazidime activity, including against ESBL-positive isolates, with a modal MIC value of 0.06 μg/ml (the highest MIC of 2 μg/ml). One exception was observed against a carbapenemase (NDM-1)-producing K. pneumoniae strain exhibiting ceftazidime-avibactam MIC results of >32 μg/ml. All baseline NF-GNB from the cIAI trial showed ceftazidime-avibactam MICs at ≤8 μg/ml, except for one P. aeruginosa isolate (32 μg/ml) expressing VIM-2 and two A. baumannii strains producing PER-1 (32 μg/ml) or OXA-23 (>32 μg/ml). Against baseline Enterobacteriaceae from cUTI patients, ceftazidime alone showed a bimodal MIC distribution and inhibited 68.3% of tested isolates at the breakpoint for susceptibility (i.e., ≤4 μg/ml). Ceftazidime-avibactam and imipenem had the same modal MIC value (0.12 μg/ml) against Enterobacteriaceae isolates causing cUTI.

**TABLE 1:** MIC results for ceftazidime, ceftazidime-avibactam, and imipenem or meropenem obtained against baseline aerobic Gram-negative pathogens recovered from the ME population

Trial	Organism	Agent^*a*^	No. of isolates at MIC (μg/ml) of:											
			≤0.03	0.06	0.12	0.25	0.5	1	2	4	8	16	32	>32
cIAI	Enterobacteriaceae^*b*^	CAZ		16	42	24	2	1	1	1		6	13	24
		CAZ-AVI	19	52	36	11	4	3	4					1^*c*^
		MER	127	1		1				1^*c*^				
	Pseudomonas spp.^*d*^	CAZ			1				6	1	2		1^*e*^	
		CAZ-AVI			1			1	6	1	1		1^*e*^	
		MER	1	2	2	2			2	1		1^*e*^		
	Acinetobacter spp.^*f*^	CAZ						1	1					2^*g*^
		CAZ-AVI					1			1			1^*g*^	1^*g*^
		MER	1		1									2^*g*^
cUTI	Enterobacteriaceae^*h*^	CAZ	2	4	22	6	3	3	3		2	5	5	8
		CAZ-AVI	8	17	28	9			1					
		IMI		17	40	6								
	P. aeruginosa	CAZ							3					
		CAZ-AVI							3					
		IMI					1	2						

a CAZ, ceftazidime; CAZ-AVI, ceftazidime-avibactam; MER, meropenem; IMI, imipenem.

b Includes 103 E. coli isolates, 16 K. pneumoniae isolates, four K. oxytoca isolates, three E. cloacae isolates, and one isolate each of Citrobacter freundii, Citrobacter braakii, Enterobacter aerogenes, and P. mirabilis.

c One NDM-1-producing K. pneumoniae isolate.

d Includes eight P. aeruginosa isolates, two P. fluorescens isolates, and one P. stutzeri isolate.

e Includes one VIM-2-producing P. aeruginosa isolate.

f Includes three A. baumannii isolates and one A. junii isolate.

g Includes one PER-1-producing A. baumannii isolate and one OXA-23-producing A. baumannii isolate.

h Includes 59 E. coli isolates, two P. mirabilis isolates, and one isolate each of Citrobacter koseri and E. cloacae.

### β-Lactamase profiles.

Totals of 38.7% (24/62) and 35.4% (46/130) of the ME patients in the cUTI and cIAI trials, respectively, had baseline pathogens that met the MIC screening criteria (i.e., were screen positive) (Tables 2 and 3). E. coli alone comprised the vast majority (88.7%) of the causative pathogens responsible for cUTI, and 38.2% met the MIC screening criteria. These isolates produced CTX-M-14 (9.5%) or CTX-M-15 (90.5%), and almost half (42.1%) of the CTX-M-15 E. coli producers also carried *bla*_OXA-1/30_. In addition, hyperproduction of the intrinsic chromosomally encoded AmpC enzyme was detected in one CTX-M-15 E. coli isolate. Except for one E. cloacae strain hyperproducing AmpC, other baseline cUTI pathogens did not meet the screening criteria (i.e., were screen negative).

**TABLE 2:** Summary of baseline aerobic Gram-negative pathogens recovered from each patient in the ME population in the cUTI trials for ceftazidime-avibactam^*a*^

Pathogen(s) in each patient (no. of patients)	Result (no. [%] of isolates)^*b*^
E. coli (55)	Screen negative (34 [61.8])
	Screen positive (21 [38.2])
	CTX-M-14 (2)
	CTX-M-15^*c*^ (11)
	CTX-M-15 + OXA-1/30 (8)
E. cloacae (1)	AmpC overexpression (1)
E. coli-C. koseri (1)	Screen negative for E. coli (1) and C. koseri (1)
E. coli-P. mirabilis (1)	CTX-M-15 for E. coli (1) and screen negative for P. mirabilis (1)
P. mirabilis (1)	Screen negative (1)
P. aeruginosa (1)	Screen negative (1)
P. aeruginosa-E. coli (2)	Screen negative for P. aeruginosa (1) and E. coli (1)
	Screen negative for P. aeruginosa (1) and CTX-M-15 + OXA-1/30 for E. coli (1)

a The cUTI trial results shown represent 62 ME patients.

b Screen negative, isolates that did not meet the MIC-based screening criteria; screen positive, isolates that met the screening criteria (i.e., ceftriaxone and/or ceftazidime MICs of ≥2 μg/ml, NF-GNB with ceftazidime MICs of ≥16 μg/ml, and Enterobacteriaceae and NF-GNB isolates exhibiting imipenem and meropenem MICs of ≥2 and ≥16 μg/ml, respectively). All Enterobacteriaceae and NF-GNB isolates had imipenem MICs of ≤0.25 and ≤2 μg/ml, respectively.

c Hyperproduction of the intrinsic AmpC enzyme was detected in one *bla*_CTX-M-15_-carrying isolate.

**TABLE 3:** Summary of baseline aerobic Gram-negative pathogens recovered from patients in the ME population in the cIAI trials for ceftazidime-avibactam^*a*^

Pathogen(s) in each patient (no. of patients)	Result (no. of isolates [% within species])^*b*^
E. coli (91)	Screen negative (58 [63.7])
	Screen positive (33 [36.3])
	CTX-M-15 (4)
	CTX-M-15 + SHV-12 (1)
	CTX-M-15 + OXA-1/30 + ACC-4 (1)
	CTX-M-15 + OXA-1/30 (14)
	CTX-M-15 + OXA-1/30 + CMY-42 (3)
	CTX-M-15 + OXA-1/30 + SHV-12 (1)
	CTX-M-15 + OXA-1/30 + SHV-2 (1)
	OXA-1/30 (1)
	SHV-12 (3)
	CMY-6 (1)
	CMY-42 (2)
	OXA-1/30 + CMY-42 (1)
K. pneumoniae (12)	Screen negative (6 [60.0])
	Screen positive (6 [40.0])
	CTX-M-15 (1)
	CTX-M-15 + OXA-1/30 (1)
	CTX-M-15 + OXA-1/30 + SHV-5 (1)
	NDM-1 + CTX-M-15 (1)
	Other screen positive^*c*^ (2)
C. braakii (1)	CTX-M-15 (1)
C. freundii (1)	Screen negative (1)
E. cloacae (3)	Screen negative (2)
	Screen positive (1)
E. aerogenes (1)	Screen negative (1)
K. oxytoca (2)	Screen negative (2)
P. mirabilis (1)	ACC-4 (1)
E. coli-K. pneumoniae (3)	Screen negative for E. coli (2) and K. pneumoniae (2)
	CTX-M-15 + OXA1/30 + SHV-31 for E. coli (1) and CTX-M-15 for K. pneumoniae (1)
E. coli-K. oxytoca (2)	Screen negative for E. coli (2) and K. oxytoca (2)
E. coli-A. baumannii (1)	CTX-M-15 for E. coli (1) and OXA-23 for A. baumannii (1)
P. aeruginosa (2)	Screen negative (1)
	VIM-2 + OXA-4 + OXA-10 (1)
P. aeruginosa-E. coli (4)	Screen negative for P. aeruginosa (4) and E. coli (4)
P. aeruginosa-A. junii (1)	Screen negative for P. aeruginosa (1) and A. junii (1)
P. aeruginosa-A. baumannii (1)	Screen negative for P. aeruginosa (1) and PER-1 for A. baumannii (1)
A. baumannii (1)	Screen negative (1)
P. fluorescens (1)	Screen negative (1)
P. fluorescens-E. coli-K. pneumoniae (1)	Screen negative for P. fluorescens (1), E. coli (1), and K. pneumoniae (1)
P. stutzeri-E. coli (1)	Screen negative for P. stutzeri (1) and E. coli (1)

a The cIAI results shown represent 130 ME patients.

b Screen negative, isolates that did not meet the MIC-based screening criteria; screen positive, isolates that met the screening criteria (i.e., ceftriaxone and/or ceftazidime MICs of ≥2 μg/ml, NF-GNB with ceftazidime MICs of ≥16 μg/ml, and Enterobacteriaceae and NF-GNB isolates exhibiting imipenem and meropenem MICs of ≥2 and ≥16 μg/ml, respectively). CMY-42 is a single-amino-acid-change (V231S) variant of CMY-2.

c Some isolates met the phenotypic MIC screening criteria, but targeted β-lactamase resistance mechanisms were not detected.

Among aerobic Gram-negative bacteria, E. coli alone represented the vast majority of baseline cIAI pathogens (70.0%), and approximately one-third (36.3%) of those isolates met the MIC screening criteria. Among these, 75.8% (25/33) carried *bla*_CTX-M-15_ alone or in combination with *bla*_SHV_ and/or *bla*_OXA-1/30_ and/or plasmid AmpC genes (i.e., *bla*_ACC_ and *bla*_CMY_ variants). Other E. coli isolates carried *bla*_OXA-1/30_ and/or *bla*_SHV_ and/or *bla*_CMY_ enzymes. Among K. pneumoniae isolates, 50.0% met the MIC screening criteria, and *bla*_CTX-M-15_, either alone or with *bla*_OXA-1/30_ and/or *bla*_SHV-5_, was present in 50.0% (3/6) of investigated bacteria. One K. pneumoniae isolate carried *bla*_NDM-1_ and *bla*_CTX-M-15_. NF-GNB (three P. aeruginosa isolates) recovered from cUTI patients were screen negative (Table 2), while three out of 15 cIAI NF-GNB met the MIC screening criteria. These three isolates were two A. baumannii isolates—one producing OXA-23 and the other PER-1—and one VIM-2-producing P. aeruginosa isolate (Table 3).

### Efficacy analysis of ceftazidime-avibactam and carbapenems in the ME population.

Table 4 summarizes the favorable response at the TOC assessment among the ME patients enrolled in the cUTI and cIAI phase 2 trials for ceftazidime-avibactam. Organisms associated with clinical or microbiological failure are presented in Table 5. Overall, favorable responses at the TOC visit were observed in 93.3 and 90.9% of ceftazidime-avibactam and comparator patients, respectively, for both trials combined when baseline MIC-based screen-negative Enterobacteriaceae isolates were recovered and in 85.7 and 80.0% of patients, respectively, when an ESBL-producing Enterobacteriaceae isolate was recovered at baseline. Favorable outcome was noted in 84.6% (22/26) of patients in the ceftazidime-avibactam arm when CTX-M-15 (alone or with additional enzymes)-producing Enterobacteriaceae was recovered at the baseline visit and in 79.2% (19/24) of patients in the carbapenem arms. In neither the ceftazidime-avibactam nor carbapenem arms of either of the trials could the microbiological or clinical failures be ascribed to high MIC of the antibacterial agent or possession of particular β-lactamase genes (Table 5).

**TABLE 4:** Summary of favorable responses at the TOC assessment among the ME patients enrolled in the cUTI and cIAI phase 2 trials for ceftazidime-avibactam

Trial	Organism(s)	Category^*a*^	Result for treatment arm by no. of patients with favorable response/total (%)^*b*^	
			Ceftazidime-avibactam	Carbapenem
cIAI and cUTI combined	Enterobacteriaceae^*c*^	Screen negative	42/45^*d*^ (93.3)	60/66 (90.9)
		Screen positive	30/35 (85.7)	24/30^*e*^ (80.0)
		CTX-M-15	22/26 (84.6)	19/24 (79.2)
		CTX-M-15 alone	8/8 (100.0)	6/10 (60.0)
		CTX-M-15 + additional ESBLs	14/18 (77.8)	17/19 (89.5)
		Other enzymes	8/9 (88.9)	5/6 (83.3)
	NF-GNB^*f*^	All	6/7^*g*^ (85.7)	9/9^*h*^ (100.0)
cIAI	Enterobacteriaceae	Screen negative	28/31 (90.3)	40/44^*i*^ (90.9)
		Screen positive	22/24 (91.7)	17/18^*i*^ (94.4)
cUTI	All^*j*^	Screen negative	15/16^*k*^ (93.8)	20/22^*l*^ (90.9)
		Screen positive	8/11 (72.7)	8/13 (61.5)
	E. coli	ST131	6/8 (75.0)	2/3 (66.7)
		Non-ST131	15/16 (93.8)	26/30 (86.7)

a Screen negative, isolates that did not meet the MIC-based screening criteria; screen positive, isolates that met the screening criteria (i.e., ceftriaxone and/or ceftazidime MICs of ≥2 μg/ml and NF-GNB with ceftazidime MICs of ≥16 μg/ml, and Enterobacteriaceae and NF-GNB isolates exhibiting imipenem and meropenem MICs of ≥2 and ≥16 μg/ml, respectively).

b Results are expressed as the number of patients with favorable responses/total number of patients in each category (percentage) and represent the ceftazidime-avibactam or imipenem-cilastatin arms for cUTI and the ceftazidime-avibactam plus metronidazole or meropenem arms for cIAI.

c Patients from whom Enterobacteriaceae pathogens were recovered at the baseline visit.

d Includes one patient with a polymicrobial infection caused by E. coli and C. koseri at the baseline visit.

e Includes one patient with a polymicrobial infection caused by aerobic Gram-negative pathogens (E. coli and K. pneumoniae).

f Patients infected with NF-GNB pathogens with or without concomitant culture of aerobic Enterobacteriaceae isolates at the baseline visit.

g Includes six patients with polymicrobial infections (4 with P. aeruginosa-E. coli, 1 with P. aeruginosa-A. baumannii, and 1 with P. stutzeri-E. coli).

h Includes five patients with polymicrobial infections (2 with P. aeruginosa-E. coli, 1 with E. coli-A. baumannii, and 1 with P. fluorescens-E. coli-K. pneumoniae).

i Includes one patient with a polymicrobial infection (E. coli-K. pneumoniae) with β-lactamase-producing pathogens and four patients with polymicrobial infections (2 with E. coli-K. pneumoniae and 2 with E. coli-K. oxytoca) with MIC-based screen-negative pathogens.

j Patients infected with Enterobacteriaceae pathogens, unless otherwise indicated.

k Includes two patients with polymicrobial infection (1 with E. coli-P. aeruginosa and 1 with E. coli-C. koseri) and one patient infected with a P. aeruginosa isolate at baseline.

l Includes two patients with polymicrobial infections (1 with E. coli-P. aeruginosa and 1 with E. coli-P. mirabilis) at baseline.

**TABLE 5:** Summary of results from patients with unfavorable response at the TOC assessment among the ME patients enrolled in the cUTI and cIAI phase 2 trials for ceftazidime-avibactam

Trial^*a*^	Arm^*b*^	Patient no.	Age (yr)	Pathogen	MIC (μg/ml)			Molecular characterization
					CAZ	CAZ-AVI	MER/IMI	
cUTI	CAZ-AVI	1	28	E. coli	1	0.12	0.12	CTX-M-14 + TEM-1
		2	36	E. coli	>32	0.25	0.12	CTX-M-15 + OXA-1 + TEM-1
		3	57	E. coli	8	0.12	0.12	CTX-M-15 + OXA-1
		4	36	P. aeruginosa	4	4	0.5	Screen negative^*c*^
	IMI	5	76	E. coli	32	0.12	0.06	CTX-M-15 + TEM-1
		6	34	E. coli	>32	0.12	0.12	CTX-M-15 + TEM-1
		7	45	E. coli	8	0.12	0.12	CTX-M-15 + TEM-1
		8	45	E. coli	0.5	0.12	0.12	Screen negative
		9	50	E. coli	16	0.12	0.12	CTX-M-15 + TEM-1
				P. mirabilis	≤0.03	≤0.03	0.25	Screen negative
		10	68	E. coli	0.12	0.06	0.12	Screen negative
		11	71	E. cloacae	>32	2	0.25	Upregulated AmpC + TEM-1
cIAI	CAZ-AVI/MTZ	1	52	E. coli	16	≤0.03	≤0.004	CTX-M-15 + OXA-1 + SHV-2
		2	40	E. coli	>32	2	0.015	CTX-M-15 + OXA-1 + CMY-42
		3	49	E. coli	0.25	0.12	0.015	Screen negative
		4	30	E. coli	0.06	≤0.03	0.008	Screen negative
		5	33	E. coli	0.12	0.12	0.015	Screen negative
	MER	6	20	E. coli	>32	0.12	0.015	CTX-M-15 + OXA-1 + SHV-12 + TEM-1
		7	82	E. aerogenes	0.12	0.12	0.03	Screen negative
		8	39	E. coli	0.12	0.06	0.015	Screen negative
		9	69	E. coli	0.12	0.06	0.015	Screen negative
		10	26	E. coli	0.12	0.06	0.015	Screen negative

a cUTI, complicated urinary tract infection; cIAI, complicated intra-abdominal infection.

b CAZ, ceftazidime; CAZ-AVI, ceftazidime-avibactam; MER, meropenem; IMI, imipenem.

c Screen negative, isolates that did not meet the MIC-based screening criteria.

^d^ Patient with polymicrobial infections (E. coli-P. mirabilis) at baseline. A molecularly unrelated (PFGE) E. coli isolate (CTX-M-15 + OXA-1) was recovered at visit 5.

^e^ A CTX-M-15-producing E. coli isolate was recovered at visit 5.

^f^ A molecularly unrelated (by PFGE) E. coli isolate (NDM-1 + CTX-M-15 + OXA-1 + CMY-2) was recovered at the follow-up visit.

Similar proportions of patients with favorable outcomes were observed for the ceftazidime-avibactam and carbapenem arms in the phase 2 cIAI trial, regardless of the presence of MIC-based screen-positive or -negative Enterobacteriaceae recovered at baseline (91.7 and 90.3% for ceftazidime-avibactam and 94.4 and 90.9% for carbapenem, respectively). Favorable outcome in the ceftazidime-avibactam arm of the phase 2 cUTI trial was less frequent (72.7%) when an isolate that met the MIC screening criteria was recovered at baseline than for patients with MIC-based screen-negative isolates (93.8%). The favorable outcome rate (61.5%) in the imipenem cUTI arm when MIC-based screen-positive Enterobacteriaceae were identified at baseline was lower than that (90.9%) for patients with a screen-negative isolate.

Overall, for both trials combined, favorable responses were documented for 85.7% (6/7) and 100.0% (9/9) of patients in the ceftazidime-avibactam and carbapenem arms, respectively, when an NF-GNB isolate was identified at baseline. In addition, favorable response rates of 75.0% (6/8) and 66.7% (2/3) were noted at the TOC for patients in the ceftazidime-avibactam and imipenem arms, respectively, of the cUTI trial, when ST131-like E. coli isolates were identified at baseline. These rates were 93.8% (15/16) and 86.7% (26/30) for the ceftazidime-avibactam and imipenem arms, respectively, when non-ST131-like E. coli isolates were recovered at baseline. Of note, three cIAI patients from the meropenem arm had carbapenemase-producing isolates. These isolates were one OXA-23 A. baumannii isolate (meropenem MIC, 16 μg/ml), one VIM-2 P. aeruginosa isolate (meropenem MIC, >16 μg/ml), and one NDM-1 K. pneumoniae isolate (meropenem MIC, 4 μg/ml) (Table 3). In addition, the patient infected with OXA-23-producing A. baumannii also had a CTX-M-15 E. coli isolate. All three patients had a favorable outcome.

## DISCUSSION

Overall, 35.2% (38.1 and 33.8% of cUTI and cIAI isolates, respectively) of all baseline Enterobacteriaceae met the MIC-based-screening criteria at the baseline visit. Previous studies have reported rates of ESBL isolates of 3.7 and 2.4% for cUTI and cIAI, respectively, in large phase 3 clinical trials (21) or 16.2% in community-acquired cIAI (22). Recent phase 3 clinical trials for ceftolozane-tazobactam reported ESBL rates in Enterobacteriaceae of 14.8 and 7.2% for cUTI and cIAI, respectively (23, 24). These rates can be influenced by geography, population, and enrollment criteria, among other factors. However, the ESBL rates reported here seem to be higher than most rates documented previously. In addition, E. coli comprised the most common pathogen in both trials, consistent with previous results (23–26), and ESBL rates among this pathogen were 36.3% among baseline E. coli isolates causing cIAI and 38.2% in those causing cUTI. However, other smaller studies have reported ESBL rates of up to 60% in E. coli isolates causing cUTI (18, 27).

Among the MIC-based screen-positive Enterobacteriaceae presented here, production of CTX-M-type enzymes, with or without the presence of OXA-1/30, was the most prevalent β-lactam resistance mechanism. Recent publications have reported the increased prevalence of CTX-M-type enzymes in Enterobacteriaceae, especially E. coli, regardless of infection source (i.e., hospital versus community acquired) (14, 15, 28). Interestingly, compared with the baseline Enterobacteriaceae isolates causing cUTI, those responsible for cIAI demonstrated a more heterogeneous β-lactamase genetic profiling. The reasons for these differences are unclear but might perhaps be attributed to geographical differences or other patient differences between trial populations.

Overall, the rate of favorable responses was 90 to 93% for both arms (ceftazidime-avibactam and carbapenems) in the aggregated data analysis (both trials combined) when an Enterobacteriaceae isolate that did not meet the screening criteria was cultured at the baseline visit. The rate of favorable responses decreased when infections were caused by isolates that met the MIC screening criteria (screen-positive isolates), with a rate for ceftazidime-avibactam (85.7%) slightly higher than that obtained for the carbapenems (80.0%). The latter overall rates were driven by those responses obtained from the cUTI trial, where the presence of a screen-positive isolate at the baseline visit predicted a less favorable outcome (72.7 and 61.5% for ceftazidime-avibactam and imipenem, respectively) compared to when a screen-negative isolate was recovered (93.8 and 90.9% for ceftazidime-avibactam and imipenem, respectively). The reasons for these findings are uncertain, although they may be related to underlying patient factors associated with acquisition of drug-resistant bacteria. The phase 2 trials were not powered to demonstrate noninferiority, and additional studies with larger numbers of patients are needed to further evaluate and confirm these results.

In this study, the number of ST131 isolates causing cUTI was relatively small (19.3% versus 80.7% for non-ST131 strains). The prevalence of ST131 among E. coli isolates varies among studies, mostly due to different organism inclusion criteria (29), and rates of ST131 isolates causing UTI of 12% have been reported in one study (30), while another study documented a rate of 30% of E. coli isolates causing pyelonephritis in Australia (31). The rates of a favorable cUTI outcome due to ST131 strains reported here were 75.0 and 66.7% for ceftazidime-avibactam and imipenem-cilastatin, respectively, while higher rates were documented when non-ST131 strains were recovered at the baseline visit (93.8 and 86.7%, respectively). These results indicate a higher treatment failure for patients infected with ST131, which corroborates a recent report published by Can et al. (31). E. coli ST131 is a multidrug-resistant (MDR) clonal group that has spread throughout the world (32). Overall, clonal expansion of ST131 is the predominant mechanism for the rising prevalence of both fluoroquinolone resistance and CTX-M-15 producers among E. coli, and consequently the ST131 E. coli population causing infections in humans (33). The dissemination of MDR strains of E. coli has challenged the empirical treatment, especially UTI, and increased morbidity and mortality (34). However, a few studies have reported similar mortality rates between infections caused by ST131 and non-ST131 strains (35, 36).

The results presented here document high rates of MIC-based screen-positive pathogens causing cUTI and cIAI, which support the current recommendations for empirical treatment of such infections (carbapenems for both cUTI and cIAI). These clinical trial results suggest that the efficacy of ceftazidime-avibactam (with metronidazole for cIAI) may be similar to that of imipenem for the treatment of cUTI or meropenem for the treatment of cIAI caused by ESBL producers, including prevalent CTX-M-15 Enterobacteriaceae.

Limitations of this study include the relatively small number of ME patients available for analysis in each arm of each trial and the even smaller numbers of patients infected by ESBL organisms. Therefore, trial results were also analyzed in aggregate to increase the number of patients for a more robust data analysis. Therefore, it is also important to mention that the patients originated from two different trial designs with distinct primary efficacy endpoints. Similarly, the association of specific E. coli lineages (ST131) with outcomes may be limited as a consequence of the small number sampled. In addition, this study evaluated aerobic Gram-negative pathogens only and did not take into consideration the presence of polymicrobial infections caused by Gram-positive bacteria and/or anaerobes, especially in the cIAI arms. Moreover, the isolates investigated here may possess β-lactam resistance mechanisms other than those screened in this study, such as other β-lactamases and/or decreased permeability.

In summary, this study shows high proportions (36.4 and 33.1% in the cUTI and cIAI trials, respectively) of aerobic Gram-negative isolates that met the screening criteria for ESBL production. All but one baseline pathogen from the cUTI trial that met the MIC screening criteria produced CTX-M enzymes, showing the dominance of CTX-M isolates causing cUTI. In contrast, a greater diversity of ESBL-encoding genes was observed among baseline isolates causing cIAI. Moreover, the efficacy of ceftazidime-avibactam was similar to that of carbapenems for treatment of cUTI and cIAI caused by ESBL organisms. However, these phase 2 clinical trials were not powered to demonstrate noninferiority, and 95% confidence intervals for the differences in responses between ceftazidime-avibactam and carbapenem treatments were expectedly wide (11, 12). Likewise, although favorable responses were similar between ceftazidime-avibactam and carbapenem for the subgroups presented here, formal statistical comparisons were not performed due to the small sample sizes and consequent large 95% confidence intervals for the treatment differences. Further and more robust analyses with a larger sample size are necessary to confirm these results, which can be expected when the phase 3 trials' results for ceftazidime-avibactam become available.

## References

[1] Jacob JT, Klein E, Laxminarayan R, Beldavs Z, Lynfield R, Kallen AJ, Ricks P, Edwards J, Srinivasan A, Fridkin S, Rasheed KJ, Lonsway D, Bulens S, Herrera R, McDonald LC, Patel J, Limbago B, Bell M, Cardo D 2013 Vital signs: carbapenem-resistant Enterobacteriaceae. MMWR Morb Mortal Wkly Rep 62:165–170. 23466435 PMC4604788

[2] Perez F, Endimiani A, Hujer KM, Bonomo RA 2007 The continuing challenge of ESBLs. Curr Opin Pharmacol 7:459–469. doi:10.1016/j.coph.2007.08.003. 17875405 PMC2235939

[3] Pitout JD, Nordmann P, Laupland KB, Poirel L 2005 Emergence of Enterobacteriaceae producing extended-spectrum β-lactamases (ESBLs) in the community. J Antimicrob Chemother 56:52–59. doi:10.1093/jac/dki166. 15917288

[4] Castanheira M, Mendes RE, Rhomberg PR, Jones RN 2008 Rapid emergence of *bla*_CTX-M_ among Enterobacteriaceae in U.S. medical centers: molecular evaluation from the MYSTIC Program (2007). Microb Drug Resist 14:211–216. doi:10.1089/mdr.2008.0827. 18707552

[5] Castanheira M, Farrell SE, Krause KM, Jones RN, Sader HS 2014 Contemporary diversity of β-lactamases among Enterobacteriaceae in the nine United States census regions and ceftazidime-avibactam activity tested against isolates producing the most prevalent β-lactamase groups. Antimicrob Agents Chemother 58:833–838. doi:10.1128/AAC.01896-13. 24247134 PMC3910895

[6] Chen L, Mathema B, Chavda KD, DeLeo FR, Bonomo RA, Kreiswirth BN 2014 Carbapenemase-producing Klebsiella pneumoniae: molecular and genetic decoding. Trends Microbiol 22:686–696. doi:10.1016/j.tim.2014.09.003. 25304194 PMC4365952

[7] Munoz-Price LS, Poirel L, Bonomo RA, Schwaber MJ, Daikos GL, Cormican M, Cornaglia G, Garau J, Gniadkowski M, Hayden MK, Kumarasamy K, Livermore DM, Maya JJ, Nordmann P, Patel JB, Paterson DL, Pitout J, Villegas MV, Wang H, Woodford N, Quinn JP 2013 Clinical epidemiology of the global expansion of Klebsiella pneumoniae carbapenemases. Lancet Infect Dis 13:785–796. doi:10.1016/S1473-3099(13)70190-7. 23969216 PMC4673667

[8] Livermore DM 2012 Fourteen years in resistance. Int J Antimicrob Agents 39:283–294. doi:10.1016/j.ijantimicag.2011.12.012. 22386741

[9] Bogan C, Kaye KS, Chopra T, Hayakawa K, Pogue JM, Lephart PR, Bheemreddy S, Lazarovitch T, Zaidenstein R, Perez F, Bonomo RA, Marchaim D 2014 Outcomes of carbapenem-resistant Enterobacteriaceae isolation: matched analysis. Am J Infect Control 42:612–620. doi:10.1016/j.ajic.2014.02.013. 24837111

[10] Drawz SM, Papp-Wallace KM, Bonomo RA 2014 New β-lactamase inhibitors: a therapeutic renaissance in an MDR world. Antimicrob Agents Chemother 58:1835–1846. doi:10.1128/AAC.00826-13. 24379206 PMC4023773

[11] Lucasti C, Popescu I, Ramesh MK, Lipka J, Sable C 2013 Comparative study of the efficacy and safety of ceftazidime/avibactam plus metronidazole versus meropenem in the treatment of complicated intra-abdominal infections in hospitalized adults: results of a randomized, double-blind, phase II trial. J Antimicrob Chemother 68:1183–1192. doi:10.1093/jac/dks523. 23391714

[12] Vazquez JA, Gonzalez Patzan LD, Stricklin D, Duttaroy DD, Kreidly Z, Lipka J, Sable C 2012 Efficacy and safety of ceftazidime-avibactam versus imipenem-cilastatin in the treatment of complicated urinary tract infections, including acute pyelonephritis, in hospitalized adults: results of a prospective, investigator-blinded, randomized study. Curr Med Res Opin 28:1921–1931. doi:10.1185/03007995.2012.748653. 23145859

[13] Clinical and Laboratory Standards Institute. 2012 M07-A9. Methods for dilution antimicrobial susceptibility tests for bacteria that grow aerobically; approved standard, 9th ed Clinical and Laboratory Standards Institute, Wayne, PA.

[14] Castanheira M, Farrell SE, Deshpande LM, Mendes RE, Jones RN 2013 Prevalence of β-lactamase encoding genes among Enterobacteriaceae bacteremia isolates collected in 26 U.S. hospitals: report from the SENTRY antimicrobial surveillance program (2010). Antimicrob Agents Chemother 57:3012–3020. doi:10.1128/AAC.02252-12. 23587957 PMC3697373

[15] Castanheira M, Mendes RE, Woosley LN, Jones RN 2011 Trends in carbapenemase-producing Escherichia coli and Klebsiella spp. from Europe and the Americas: report from the SENTRY antimicrobial surveillance programme (2007-09). J Antimicrob Chemother 66:1409–1411. doi:10.1093/jac/dkr081. 21421581

[16] Castanheira M, Costello SE, Woosley LN, Deshpande LM, Davies TA, Jones RN 2014 Evaluation of clonality and carbapenem resistance mechanisms among Acinetobacter baumannii-Acinetobacter calcoaceticus complex and Enterobacteriaceae isolates collected in European and Mediterranean countries and detection of two novel β-lactamases, GES-22 and VIM-35. Antimicrob Agents Chemother 58:7358–7366. doi:10.1128/AAC.03930-14. 25267671 PMC4249534

[17] Castanheira M, Deshpande LM, Costello A, Davies TA, Jones RN 2014 Epidemiology and carbapenem resistance mechanisms of carbapenem-non-susceptible Pseudomonas aeruginosa collected during 2009-2011 in 14 European and Mediterranean countries. J Antimicrob Chemother 69:1804–1814. doi:10.1093/jac/dku048. 24603963

[18] Picozzi SC, Casellato S, Rossini M, Paola G, Tejada M, Costa E, Carmignani L 2014 Extended-spectrum β-lactamase-positive Escherichia coli causing complicated upper urinary tract infection: urologist should act in time. Urol Ann 6:107–112. doi:10.4103/0974-7796.130536. 24833818 PMC4021646

[19] Pitout JD, Laupland KB 2008 Extended-spectrum β-lactamase-producing Enterobacteriaceae: an emerging public-health concern. Lancet Infect Dis 8:159–166. doi:10.1016/S1473-3099(08)70041-0. 18291338

[20] Dhanji H, Doumith M, Clermont O, Denamur E, Hope R, Livermore DM, Woodford N 2010 Real-time PCR for detection of the O25b-ST131 clone of Escherichia coli and its CTX-M-15-like extended-spectrum β-lactamases. Int J Antimicrob Agents 36:355–358. doi:10.1016/j.ijantimicag.2010.06.007. 20691573

[21] Kaniga K, Flamm R, Tong SY, Lee M, Friedland I, Redman R 2010 Worldwide experience with the use of doripenem against extended-spectrum β-lactamase-producing and ciprofloxacin-resistant Enterobacteriaceae: analysis of six phase 3 clinical studies. Antimicrob Agents Chemother 54:2119–2124. doi:10.1128/AAC.01450-09. 20211892 PMC2863686

[22] Jean SS, Ko WC, Xie Y, Pawar V, Zhang D, Prajapati G, Mendoza M, Kiratisin P, Ramalheira E, Castro AP, Rosso F, Hsueh PR 2014 Clinical characteristics of patients with community-acquired complicated intra-abdominal infections: a prospective, multicentre, observational study. Int J Antimicrob Agents 44:222–228. doi:10.1016/j.ijantimicag.2014.05.018. 25106073

[23] Wagenlehner FM, Umeh O, Steenbergen J, Yuan G, Darouiche RO 2015 Ceftolozane-tazobactam compared with levofloxacin in the treatment of complicated urinary-tract infections, including pyelonephritis: a randomised, double-blind, phase 3 trial (ASPECT-cUTI). Lancet 385:1949–1956. doi:10.1016/S0140-6736(14)62220-0. 25931244

[24] Solomkin J, Hershberger E, Miller B, Popejoy M, Friedland I, Steenbergen J, Yoon M, Collins S, Yuan G, Barie PS, Eckmann C 2015 Ceftolozane/tazobactam plus metronidazole for complicated intra-abdominal infections in an era of multidrug resistance: results from a randomized, double-blind, phase 3 trial (ASPECT-cIAI). Clin Infect Dis 60:1462–1471. doi:10.1093/cid/civ097. 25670823 PMC4412191

[25] Naber KG, Llorens L, Kaniga K, Kotey P, Hedrich D, Redman R 2009 Intravenous doripenem at 500 milligrams versus levofloxacin at 250 milligrams, with an option to switch to oral therapy, for treatment of complicated lower urinary tract infection and pyelonephritis. Antimicrob Agents Chemother 53:3782–3792. doi:10.1128/AAC.00837-08. 19581455 PMC2737884

[26] Lucasti C, Jasovich A, Umeh O, Jiang J, Kaniga K, Friedland I 2008 Efficacy and tolerability of IV doripenem versus meropenem in adults with complicated intra-abdominal infection: a phase III, prospective, multicenter, randomized, double-blind, noninferiority study. Clin Ther 30:868–883. doi:10.1016/j.clinthera.2008.04.019. 18555934

[27] Qiao LD, Chen S, Yang Y, Zhang K, Zheng B, Guo HF, Yang B, Niu YJ, Wang Y, Shi BK, Yang WM, Zhao XK, Gao XF, Chen M, Tian Y 2013 Characteristics of urinary tract infection pathogens and their in vitro susceptibility to antimicrobial agents in China: data from a multicenter study. BMJ Open 3:e004152. doi:10.1136/bmjopen-2013-004152. PMC3863131 24334199

[28] Hayakawa K, Gattu S, Marchaim D, Bhargava A, Palla M, Alshabani K, Gudur UM, Pulluru H, Bathina P, Sundaragiri PR, Sarkar M, Kakarlapudi H, Ramasamy B, Nanjireddy P, Mohin S, Dasagi M, Datla S, Kuchipudi V, Reddy S, Shahani S, Upputuri V, Marrey S, Gannamani V, Madhanagopal N, Annangi S, Sudha B, Muppavarapu KS, Moshos JA, Lephart PR, Pogue JM, Bush K, Kaye KS 2013 Epidemiology and risk factors for isolation of Escherichia coli producing CTX-M-type extended-spectrum β-lactamase in a large U.S. medical center. Antimicrob Agents Chemother 57:4010–4018. doi:10.1128/AAC.02516-12. 23752516 PMC3719715

[29] Rogers BA, Sidjabat HE, Paterson DL 2011 Escherichia coli O25b-ST131: a pandemic, multiresistant, community-associated strain. J Antimicrob Chemother 66:1–14. doi:10.1093/jac/dkq415. 21081548

[30] Can F, Azap OK, Seref C, Ispir P, Arslan H, Ergonul O 2015 Emerging Escherichia coli O25b/ST131 clone predicts treatment failure in urinary tract infections. Clin Infect Dis 60:523–527. doi:10.1093/cid/ciu864. 25378460

[31] Kudinha T, Johnson JR, Andrew SD, Kong F, Anderson P, Gilbert GL 2013 Escherichia coli sequence type 131 as a prominent cause of antibiotic resistance among urinary Escherichia coli isolates from reproductive-age women. J Clin Microbiol 51:3270–3276. doi:10.1128/JCM.01315-13. 23885001 PMC3811657

[32] Nicolas-Chanoine MH, Blanco J, Leflon-Guibout V, Demarty R, Alonso MP, Canica MM, Park YJ, Lavigne JP, Pitout J, Johnson JR 2008 Intercontinental emergence of Escherichia coli clone O25:H4-ST131 producing CTX-M-15. J Antimicrob Chemother 61:273–281. 18077311 10.1093/jac/dkm464

[33] Banerjee R, Johnson JR 2014 A new clone sweeps clean: the enigmatic emergence of Escherichia coli sequence type 131. Antimicrob Agents Chemother 58:4997–5004. doi:10.1128/AAC.02824-14. 24867985 PMC4135879

[34] Ben-Ami R, Rodriguez-Bano J, Arslan H, Pitout JD, Quentin C, Calbo ES, Azap OK, Arpin C, Pascual A, Livermore DM, Garau J, Carmeli Y 2009 A multinational survey of risk factors for infection with extended-spectrum β-lactamase-producing Enterobacteriaceae in nonhospitalized patients. Clin Infect Dis 49:682–690. doi:10.1086/604713. 19622043

[35] Chung HC, Lai CH, Lin JN, Huang CK, Liang SH, Chen WF, Shih YC, Lin HH, Wang JL 2012 Bacteremia caused by extended-spectrum β-lactamase-producing Escherichia coli sequence type ST131 and non-ST131 clones: comparison of demographic data, clinical features, and mortality. Antimicrob Agents Chemother 56:618–622. doi:10.1128/AAC.05753-11. 22123694 PMC3264214

[36] Lopez-Cerero L, Navarro MD, Bellido M, Martin-Pena A, Vinas L, Cisneros JM, Gomez-Langley SL, Sanchez-Monteseirin H, Morales I, Pascual A, Rodriguez-Bano J 2014 Escherichia coli belonging to the worldwide emerging epidemic clonal group O25b/ST131: risk factors and clinical implications. J Antimicrob Chemother 69:809–814. doi:10.1093/jac/dkt405. 24123431

